# Growth adaptability and stability in *Catalpa bungei* clones: the role of genetics and environment

**DOI:** 10.48130/forres-0025-0003

**Published:** 2025-01-22

**Authors:** Yao Xiao, Zhengde Wang, Junhui Wang, Huiling Yun, Juanjuan Ling, Wenji Zhai, Kun Zhao, Xiaochi Yu, Wenjun Ma

**Affiliations:** 1 State Key Laboratory of Tree Genetics and Breeding, Key Laboratory of Forest Tree Breeding, State Forestry and Grassland Administration, Catalpa Bungei National Innovation Alliance, Research Institute of Forestry, Chinese Academy of Forestry, Beijing 100091, China; 2 Xiaolongshan Research Institute of Forestry, Tianshui 741032, China; 3 Nanyang Forestry Science Research Institute, Nanyang 473001, China; 4 Luoyang Academy of Agriculture and Forestry, Luoyang 471023, China

**Keywords:** *Catalpa bungei*, Environment variation, Genetic variation, Genotype-by-environment interactions, Stability and productivity

## Abstract

Understanding genotype, environment, and genotype-by-environment (G × E) interactions is vital for effective forest breeding. The *Catalpa bungei*, valued for its rapid growth and high-quality wood, exhibits uncertain genetic variation in growth across diverse ecological conditions. To clarify this, we measured the growth traits of clones over several years at multiple sites to evaluate the effects of genetics and environment on growth. The results indicate that growth traits exhibit significant genetic differences and high repeatability, and the significant G × E interaction highlights the importance of site-specific tree selection. Correlation and regression analysis indicated that MCMT was positively correlated with DBH, whereas DD < 18 was negatively correlated with DBH. TD and CMD showed positive correlations with height and volume. Multivariate regression trees (MRT) analysis showed that clones thrived under specific conditions: TD > 26.65 °C with MCMT > 0.1 °C and CMD > 520.5. Mantel analysis results indicated that TD is the main factor driving the G × E of DBH. To identify clones well-suited for targeted cultivation and stability in various regions, we estimated BLUP values for clone growth and applied BLUP-GGE to assess the yield and stability of 5-year height, 9-year DBH, and 5-year volume. Clone 1-1 was selected for its high and stable DBH, with a 6.22% genetic gain. Clone 22-03 was selected for its high and stable volume, with a 12.11% gain. These elite clones are anticipated to boost *C. bungei* plantation productivity and are likely to be cultivated and promoted across multiple regions.

## Introduction

Forest productivity is the goal of artificial forest development. Factors that determine forest productivity include external site conditions, climatic conditions, and management measures, as well as internal genetic characteristics of tree species. Although China's artificial forest area ranks first in the world, the forest stock volume is only 84% of the world's average level, and the per-unit area stock volume is only about 50% of that of natural forests^[1]^. The confusion of artificial forest seed sources, unclear genetic control of traits, and the blind selection of unsuitable tree species for the site are important factors affecting their productivity. The genetic control of growth variation is the basis for genetic improvement and selection of superior varieties^[2]^, and no significant genetic differences between genotypes or insufficient heritability of the traits can hinder genetic improvement. Therefore, genetic evaluation is a necessary step in the genetic improvement of forest trees.

The phenotypes of trees are the result of a combination of genotype, environment, and genotype and environment (G × E) interaction. When genetic effects are significant, accurately assessing the G × E interaction is essential for formulating correct breeding strategies, as there are often substantial differences in the environmental response patterns of the same trait among different tree species or even among different genotypes^[3]^. Numerous studies have shown that there are often significant G × E interactions for *Pinus elliottii*^[4]^, *Liriodendron tulipifera*^[5]^, *Pinus massoniana*^[6]^, *Picea koraiensis*^[7]^, and *Populus tomentosa*^[8]^. A study showed the specific environmental factors, such as topography and soil conditions^[9]^ as well as temperature and humidity differences caused by altitude^[10,11]^, that may be the main drivers of G × E effects. Thus, in the process of selecting and breeding superior tree genotypes, it is essential for evaluating the G × E interactions caused by differences in the geographical environment through multi-location trials to determine the yield levels and stability of genotypes^[12,13]^. Furthermore, a deep understanding of the environmental factors driving the G × E interaction is greatly beneficial for the selection of suitable cultivation areas for trees and the formulation of breeding strategies.

G × E interactions complicate genotypic variance and affect the estimation of genetic parameters, which can lead to bias in the ranking of families or individual plants. Therefore, genotype performance in multiple environments is unpredictable and it is difficult to identify the best-performing genotype for a given locus^[14]^ , how to accurately assess G × E interactions is crucial for subsequent selection and promotion of good forest tree species. To be able to accurately evaluate the degree of genotype-environment interactions for forest tree traits, several analytical methods have been proposed. From the early joint regression-based stability analysis^[15]^, Type-B genetic correlation^[16]^, GGE double-labeling^[17]^, Harmonic Mean of Relative Performance of Genotypic Values (HMRPGV) method^[18]^, AMMI analysis^[19]^ up to the recent combined BLUP-GGE analysis^[20]^, the methodologies have been continuously refined. Among them, the BLUP-GGE analysis combines GGE with best linear unbiased prediction (BLUP) analysis and the use of BLUP values for each genotype at each locus, avoiding the constraints of fixed-effects models, homogeneity of test loci, and missing data^[20,21]^. To date, the BLUP-GGE biplot analysis has become an effective and convenient method for evaluating the genotype by environment (G × E) effects in forest trees^[6,7,22]^.

*Catalpa bungei* is a tree belonging to the *Catalpa* genus in the family Bignoniaceae. It has a straight trunk and superior wood quality and serves as an excellent material for industrial lumber and furniture, holding significant economic value. Therefore, reducing the cultivation period of plantation forests of *C. bungei* and enhancing the timber yield can significantly augment the supply to the industrial sector, thereby mitigating the supply pressure in the timber market. In the earlier study, we have already found that the *Catalpa* clones exhibit significant G × E interactions in growth across different years^[23]^. However, the impact of geographical environmental differences on the growth of *Catalpa* clones and the magnitude of G × E interactions under multi-environmental conditions have not yet been assessed. The objectives of this study were: (1) to evaluate the impact of genetic factors on the growth of clones and to clarify the potential for genetic improvement; (2) to analyze the main environmental factors that affect the growth of clones and drive the G × E effects; (3) to explore the effects of G × E interactions on growth traits and exactly assess the stability, and productivity of clones under different environments; and (4) use BLUP-GGE biplot analysis to identify the dominant clones that grow rapidly under a wide range of environmental conditions and those best suited to specific environments. The results of this study can help formulate better genetic improvement strategies for *C. bungei*.

## Materials and methods

### Test sites and conditions

**Figure 1:**
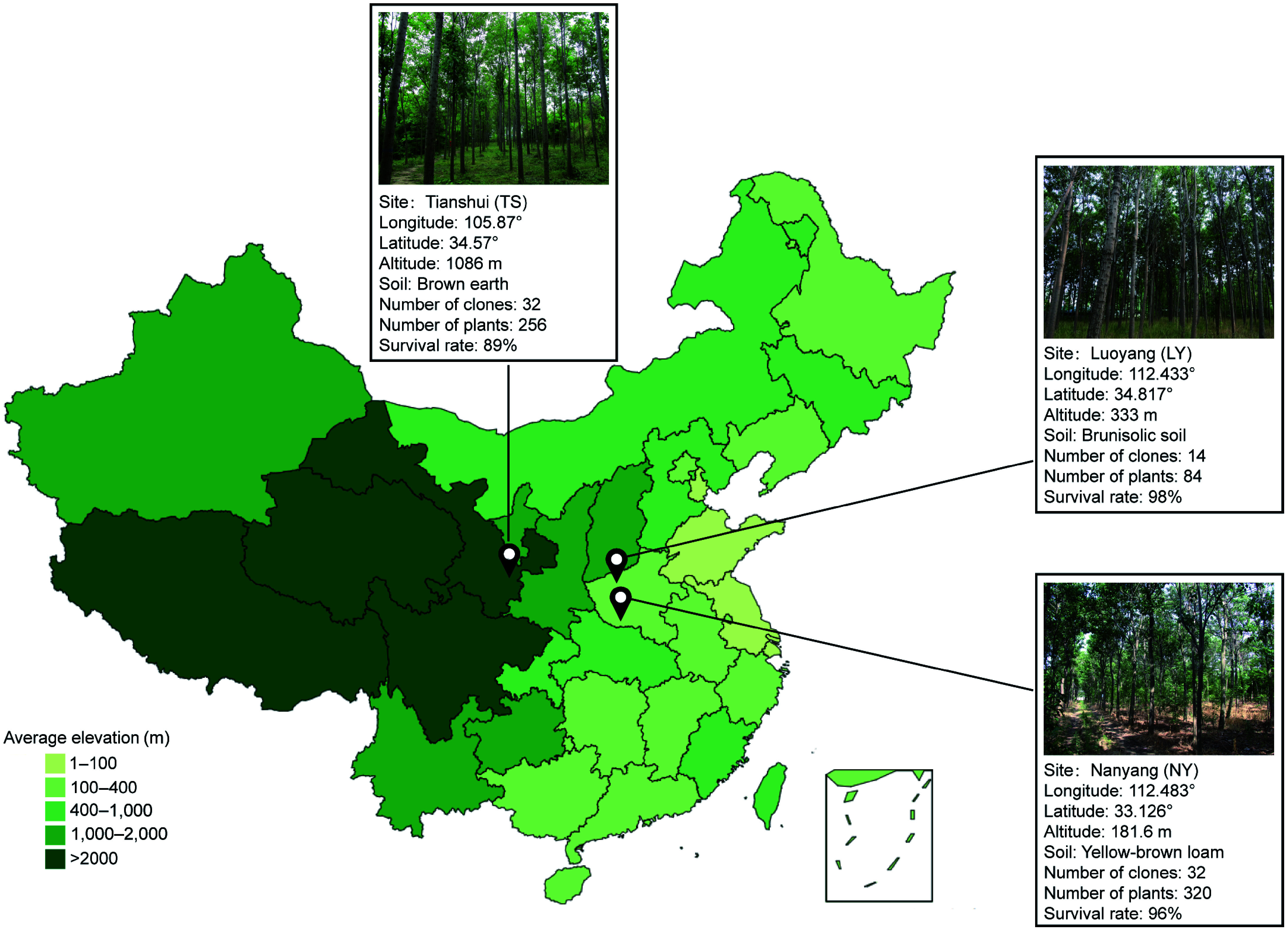
The geographical location and basic information of the three test forests.

Three test sites were designed for this experiment, located in Tianshui (TS), Gansu, Luoyang (LY), Henan, and Nanyang (NY). Henan, where TS has a temperate monsoon climate, LY has a temperate continental monsoon climate and NY has a monsoon continental humid semi-humid climate. The geographical location, altitude, and soil condition of the sites are shown in Fig. 1. Climatic data for the test sites were obtained from ClimateAP^[24]^.

### Test materials and design

A total of 32 clones were tested in this experiment, among which 23 clones were from the *C. bungei* hybrida controlled pollination, and nine were from the *C. bungei* superior tree-free pollination. The genetic background of test materials was shown in Supplemental Table S1, among which 14 clones were preserved in LY. The three sites were completely randomized block designs, among which LY had a single plant plot with six replicates, NY had a two-plant plot with five replicates, and TS had a two-plant plot with four replicates. At each site, tree height and DBH were measured at the end of the annual vegetative period when the trees were between 1 and 5 years old, and DBH was measured at the end of the annual vegetative period when the trees were between 6 and 9 years old. Due to natural deaths, missing records, and other factors, the number of plants investigated at each site was inconsistent, the survival rate of clonal lines is detailed in Fig. 1. The experimental forests at the three sites were all established in the spring of 2009.

### Estimating of variance component and genetic parameters

Estimate the volume of timber according to the formula:

$ V=0.000050479055\times {DBH}^{1.9085054}\times {Height}^{0.99076507} $

The analysis of variance and multiple comparison analyses were carried out in R software version 4.1.3. The lme4-R software package was used to estimate the genetic parameters of each clone at different sites, including the best linear unbiased estimation (BLUP), variance components of genetic effects, and repeatability. A single-site analysis was used to investigate trait differences at different age points and sites, using a general linear mixing model as follows:

$ {y}_{ijk}=\mu +{B}_{j}+{C}_{i}+{e}_{ijk} $

Where $ {y}_{ijk} $ is the observed value of the *k*-th individual of the *i*-th clonal in block j; μ is the average of the population; $ {B}_{j} $ is the fixed effect of the *j*-th block, $ {C}_{i} $ is the random effect of the *i*-th clone, NID (normally independent distribution) (0, $ {\sigma }_{c}^{2} $), $ {e}_{ijk} $ is the random residual effect of the *k*-th individual of the ith clonal in the *j*-th block, NID (0, $ {\sigma }_{e}^{2} $).

To explore environment (site) and G × E effects across sites, the following equation for multi-site analysis was used as follows:

$ {y}_{ijks}=\mu +{S}_{s}+{C}_{i}+B{S}_{  js}+C{S}_{is}+{e}_{ijks} $

Where $ {y}_{ijks} $ is the observed value of the *k*-th individual of the *i*-th clone in the *j*-th block at the *s*-th site, and μ is the average value of the population. $ {S}_{s} $ is the fixed effect at site S, $ {C}_{i} $ is the random effect of the *i*-th clone NID (0, $ {\sigma }_{c}^{2} $), $ B{S}_{  js} $ is the fixed effect at site S of block j, NID (0, $ {\sigma }_{CB}^{2} $), $ C{S}_{is} $ is the random effect at site S of clonal *i*. NID (0, $ {\sigma }_{cs}^{2} $), $ {e}_{ijks} $ is the random residual effect of the *k*-th individual of the *i-*th clone in the *j*-th block of the *s*-th site, NID (0, $ {\sigma }_{e}^{2} $).

Using the membership function method to standardize the heights, diameters at breast height (DBH), and volume of clones over multiple years, the sum of the standardized indicators for each trait per year is taken as the comprehensive evaluation index, and this parameter is used for ranking. The specific formula is as follows:

$ {X'_i}=\dfrac{{X}_{i}-{X}_{min}}{{X}_{max}-{X}_{min}} $

where $ {X'_{i}} $ is the normalized value of the indicator, $ {X}_{i} $ is the value of the trait for each clone, and $ {X}_{min} $ and $ {X}_{max} $ are the minimum and maximum value of each trait over all the clones, respectively.

The formula of the phenotypic coefficient of variation is as follows:

$ CV=\dfrac{S  D}{\overline{X}} $

Where *CV* is the phenotypic coefficient of variation, $ \overline{X} $ is the mean value of the trait at one age, *SD* is the standard deviation of traits.

The formula of the genetic variation coefficient is as follows:

$ GCV=\dfrac{\sqrt{{\sigma }_{g}^{2}}}{\overline{X}}\times 100 $

Where *GCV* is genetic variation coefficient, $ {\sigma }_{g}^{2} $ is genetic variance, $ \overline{X} $ is the mean of traits.

The formula of the environmental variation coefficient is expressed as follows:

$ ECV=\dfrac{\sqrt{{\sigma }_{e}^{2}}}{\overline{X}}\times 100 $

Where *ECV* is the environmental variation coefficient, $ {\sigma }_{e}^{2} $ is environmental variance, $ \overline{X} $ is the mean of traits.

The formula for calculating the repeatability of a single site is:

$ R={\sigma }_{C}^{2}/({\sigma }_{C}^{2}+\dfrac{{\sigma }_{e}^{2}}{K}) $

Where *R* is repeatability; $ {\sigma }_{C}^{2} $ is the variance components of the clonal effect and *K* is the harmonic number of the repeated tests.

The formula for calculating the repeatability of multi-sites is:

$ R={\sigma }_{C}^{2}/({\sigma }_{C}^{2}+\dfrac{{\sigma }_{SC}^{2}}{S}+\dfrac{{\sigma }_{e}^{2}}{S  BN}) $

Where *R* is repeatability; $ {\sigma }_{C}^{2} $ is the variance component of the clonal effect; $ {\sigma }_{SC}^{2} $ is the variance component of the interaction between clone and site, $ {\sigma }_{e}^{2} $ is the random error; *S* is the number of sites, *B* is the number of blocks, and *N* is the number of individual plants tested per block.

Type B correlations are genetic correlations between different environments and the type B genetic correlation coefficient formula:

$ {r}_{b}={\sigma }_{C}^{2}/({\sigma }_{C}^{2}+{\sigma }_{CS}^{2}) $

The following formula was used to calculate genetic gain:

$ \Delta G=\dfrac{S\times R}{\overline{X}} $

Where *R* is repeatability from multi-site tests; *S* is the selection differential and $ \overline{X} $ is the population mean.

### Multivariate regression tree (MRT) analysis

MRT is a machine learning technique that extends the univariate regression tree. It can handle multiple response variables with nonlinear relationships and missing values and is used to analyze complex ecological data, especially to explore, describe, and predict the relationships between species data and environmental characteristics^[25,26]^. MRT analysis was conducted using the R package mvpart^[27]^. A 10-fold cross-validation procedure is performed to determine the optimal tree size and to avoid model overfitting (Supplementary Fig. S1). The detailed validation mode of MRT is referenced in a previous study^[28]^.

### Correlation analysis

The analysis of environmental factors and traits was performed using the Spearman correlation analysis using SPSS 22.0 software. The absolute value of the differences in environmental factors between different regions each year was calculated as the change in environmental factors. The strength of the correlation between the change in environmental factors and the B-type genetic correlation of traits between corresponding regions was used to measure the impact of environmental factor changes on the G × E effect. The Mantel correlation analysis of trait genetic correlations and environmental factor variables was conducted using the vegan and ggcor packages in the R language.

### Genotype by environment (G × E) interaction analysis and selection of excellent clones

GGE biplots were constructed from the first two principal components (PC1 and PC2) derived by subjecting the environment-centered the BLUP value of tree height and volume at 5 years old, DBH at 9 years old of each clone at each site to singular-value decomposition. The following model was used to construct the GGE biplot^[21]^:

$ {y}_{ij}=\mu +{E}_{j}+\sum _{K=1}^{K}{b}_{ik}{z}_{jk}+{\varepsilon }_{ij} $

where $ {y}_{ij} $ is the yield of the *i* genotype in the *j*-th environment, μ is the overall mean, $ {E}_{j} $ is the effective value of the *j* environment, the genotype effect and G × E was decomposed into *K* factors multiplied together, $ {b}_{ik} $ is the variety score, $ {\varepsilon }_{ij} $ is the environment score, both are the results of principal component analysis of G + G × E, and $ {\varepsilon }_{ij} $ is the experimental error.

The GGEBiplotGUI package of R soft was used to plot GGE biplots, with the parameters set by selecting 1 for Sdt deviation, 1 for centered G + GE, and 1 or 2 for SVP eigenvalues depending on the type of plot.

## Results

### Growth trait variations and influencing effects of *Catalpa bungei* clones

**Figure 2:**
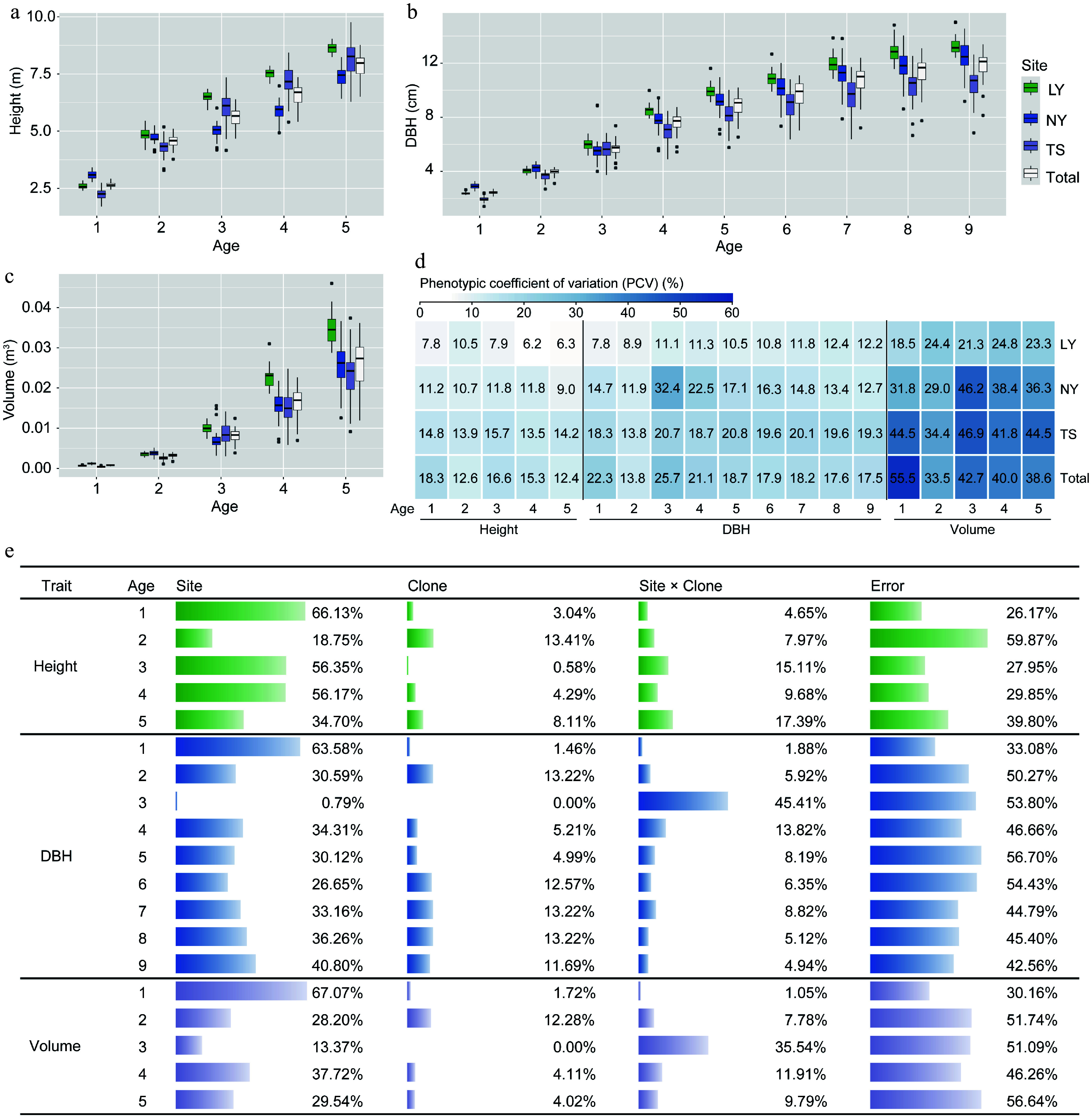
Analysis of variation in growth traits of clones under different environments. (a)−(c) Comparative differences in tree height, DBH, and volume among clones in the LY, NY, TS, and across three sites. (d) Phenotypic variation coefficients of clonal growth traits. (e) Analysis of the proportion of variance components influenced by environmental (Site), genetic (Clone), and G × E (Site × Clone) effects on clonal growth traits.

To clarify the main effects influencing the growth of clones, a study was conducted to evaluate the strength of the effects of genotype, environment, and the interaction between genotype and environment on the growth of clones using mixed linear models. It was found that significant differences in tree height and volume at 1−5 years old, and DBH at 1−9 years old were observed among sites and clones, respectively, except DBH and volume in the first year and DBH in the sixth year, all other traits exhibited significant G × E effects (Supplementary Table S2). After the third year, the height, DBH, and volume of clones grown in LY were significantly higher than those in NY and TS. However, the coefficients of variation for growth traits in LY clones (height: 6.2%−10.5%, DBH: 7.8%−12.4%, and volume: 18.5%−24.8%) were lower than those in NY (height: 9.0%−11.8%, DBH: 11.9%−32.4%, volume: 29.0%−46.2%) and TS (height: 13.5%−15.7%, DBH: 13.8%−20.8%, volume: 34.4%−46.9%) clones (Fig. 2a−d). Among the three traits, volume had the largest coefficient of variation (Fig. 2d). The results of the random effect variance components indicated that overall, differences in location environments contributed the most to the phenotypic variation of clones, while genetic effects had a smaller impact (height: 0.58%−13.41%, DBH: 0.00%−13.22%, volume: 0.00%−12.28%) (Fig. 2e), but the fixed effect analysis showed that the growth variation caused by genetics reached a significant level (Supplementary Table S2). Furthermore, the G × E for growth traits was small only in the first year, and increased in subsequent years, with the G × E variance component accounting for as much as 45.41% and 35.54% of the variation in DBH and volume, respectively, in the third year (Fig. 2e). The results suggest that the genetic improvement of *C. bungei* clones must be based on the selection of suitable clones according to specific environments.

### Analysis of genetic effects on the growth variation of clones

**Figure 3:**
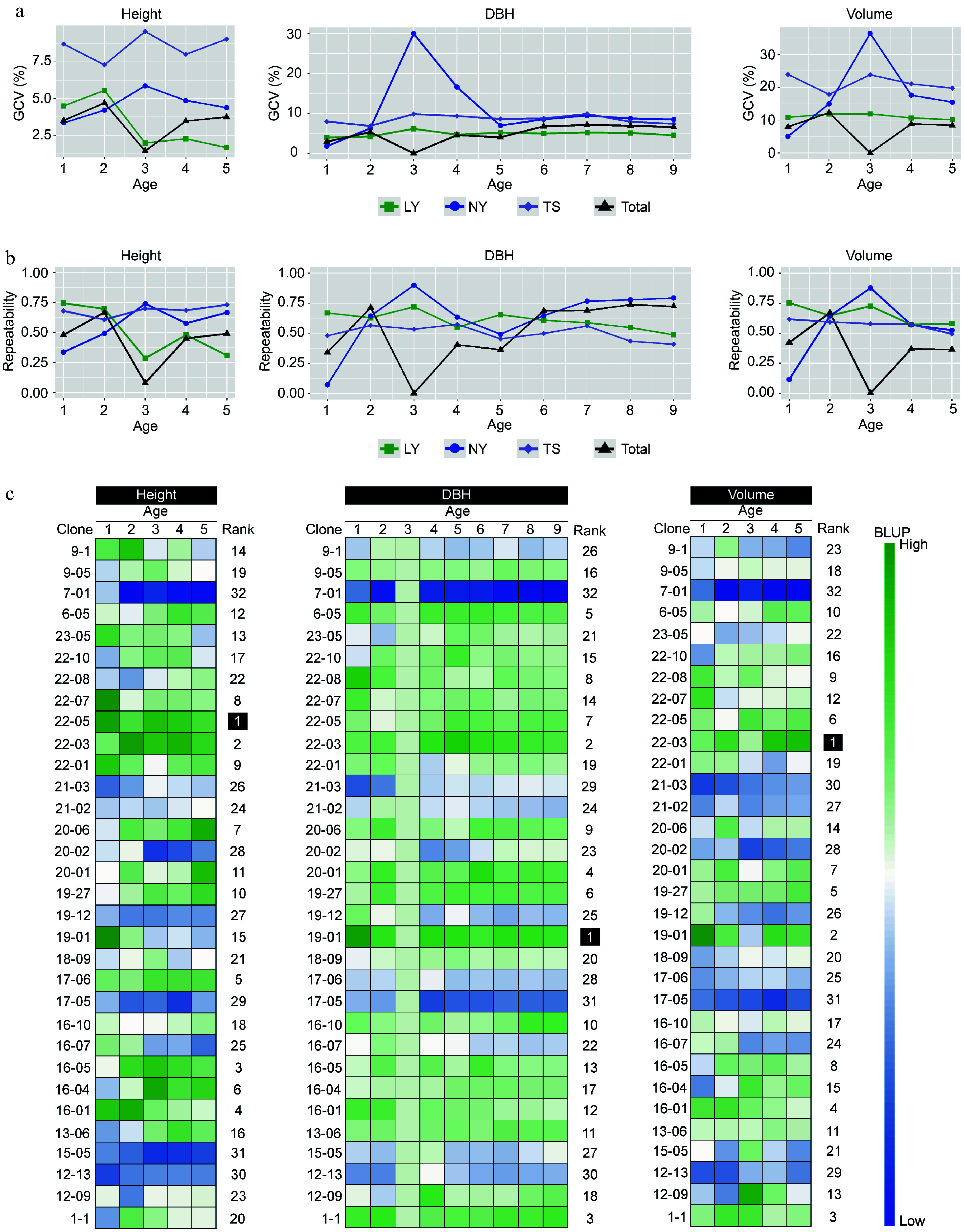
The impact of genetic effects on the growth of clones. (a) The genetic variation coefficients of multi-year growth traits of clones in different regions. (b) The repeatability of multi-year growth traits of clones in different regions. (c) The BLUP values of clonal growth traits estimated by the multi-site mixed-effects model, with Rank representing the comprehensive ranking of the membership function for a single trait's multi-year BLUP values of the same clone.

To assess the foundation for genetic improvement of clones, the study analyzed the genetic variation coefficients and repeatability of traits for the clones. The results indicated that the genetic variation in volume was greater than that in height and DBH. The genetic variation in height of clones in the TS was approximately 8%, exceeding that in the LY and NY. After growth stabilization, the genetic variation in DBH of clones could reach 5% to 10%, and the genetic variation in volume could reach 10% to 20%. Notably, in NY, the genetic variation in DBH and volume in the third year significantly increased to over 30%, but the combined genetic variation for DBH and volume at the three sites dropped to 0. The enhancement of G × E effects is the reason for this result (Fig. 3a). The results on trait repeatability showed that as the clones grew and became more stable, the repeatability of traits gradually approached stability. According to the estimates from the last year of the traits, the repeatability of height in the LY was relatively low, around 0.30, while DBH and volume had moderate repeatability of approximately 0.50 to 0.60. Clones in the NY and TS had moderately high repeatability for growth traits, ranging from 0.40 to 0.75. The multi-site repeatability estimation indicated that the repeatability of DBH in the ninth year was relatively high, reaching 0.72 (Fig. 3b). In summary, the genetic variation in clones is rich, trait variation is strongly controlled by genetics, and there is a good foundation for genetic improvement. Good improvement effects can be achieved through the selection of superior clones. To evaluate the differences in breeding value among clones, we first estimated the BLUP values for growth traits. And then using the membership function method to calculate the comprehensive assessment values of clonal growth traits indicated that clones 22-05, 19-01, and 22-03 were the top-ranked clones for height, DBH, and volume BLUP values, respectively, and can be preliminarily considered as potential candidates for selection (Fig. 3c).

### Environmental effects have a determining role in the growth of *C. bungei* clones

**Figure 4:**
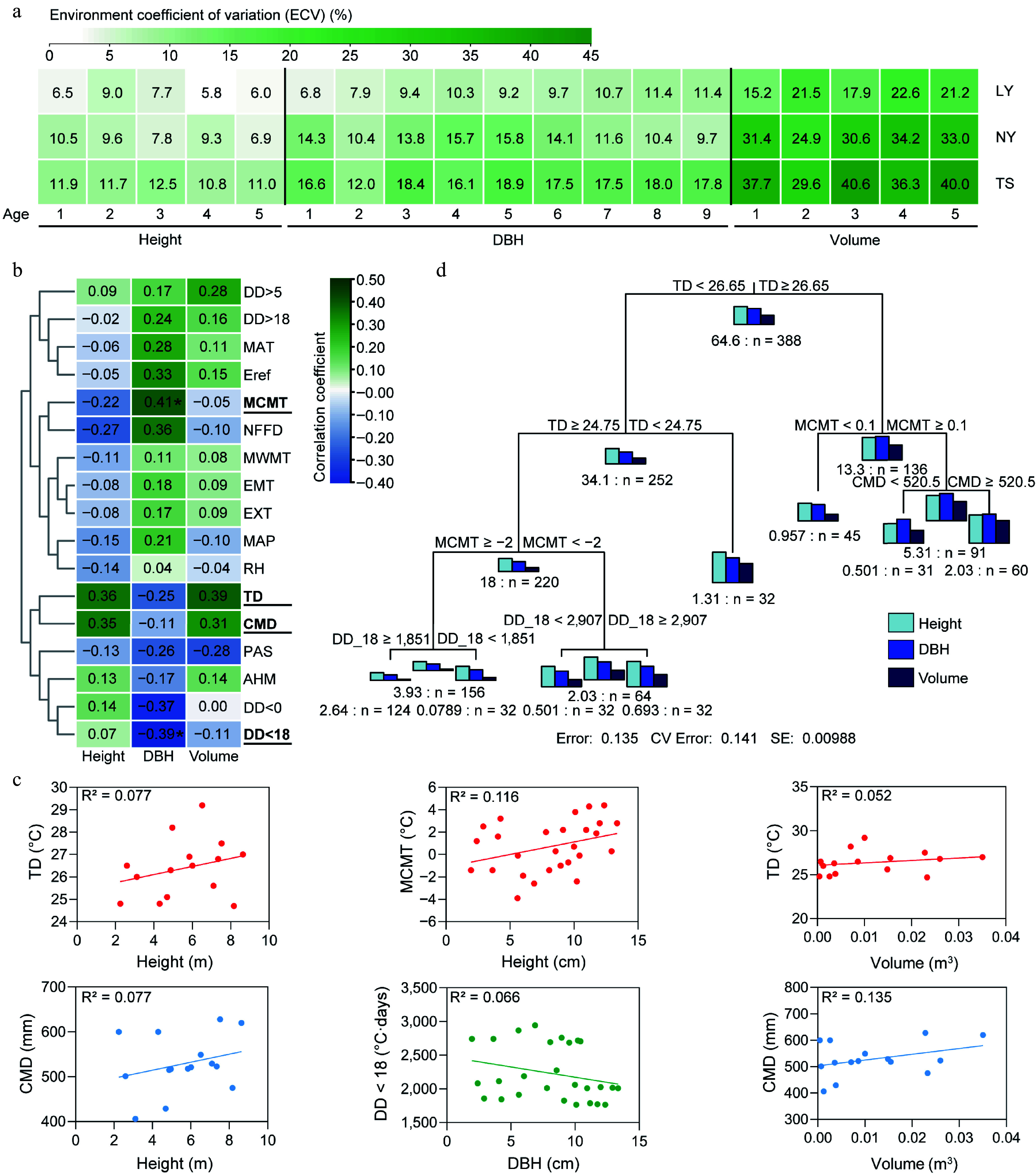
The impact of environment on the growth of clones. (a) Environmental variation coefficients of clonal growth across multiple years and sites. (b) Spearman correlation coefficients between environmental factors and growth traits. (c) Linear regression analysis of height, DBH, and volume with key environmental factors. (d) Multivariate regression tree analysis (MRT) of the comprehensive impact of the environment on the tree height, DBH, and volume of clones, with the number preceding 'n' indicating the sum of squared distances for the parameter, and 'n' representing the number of individuals in the subclass. MAT: Mean annual temperature (°C); MWMT: Mean warmest month temperature (°C); MCMT: Mean coldest month temperature (°C); TD: Temperature difference between MWMT and MCMT, or continentality (°C); MAP: Mean annual precipitation (mm); AHM: Annual heat/moisture index (MAT+10)/(MAP/1,000)); DD < 0: Degree-days below 0°C, chilling degree-days; DD > 5: Degree-days above 5 °C, growing degree-days; DD < 18: Degree-days below 18 °C, heating degree-days; DD > 18 Degree-days above 18 °C, cooling degree-days; NFFD: The number of frost-free days; PAS: Precipitation as snow between August in the previous year and July in current year (mm); EMT: Extreme minimum temperature over 30 years (°C); EXT: Extreme maximum temperature over 30 years (°C); Eref: Hargreaves reference evaporation (mm); CMD: Hargreaves climatic moisture deficit (mm); RH: Relative humidity (%).

To assess the impact of the environment on the growth of clones, we first estimated the environmental variation coefficients for growth traits at three sites. The results showed that the environmental variation coefficients for height (10.8%−12.5%), DBH (12.0%−18.8%), and volume (29.6%−40.6%) in the TS were all greater than those in the LY (height: 5.8%−9.0%, DBH: 6.8%−11.4%, volume: 15.2%−22.6%) and NY (height: 6.9%−10.5%, DBH: 9.7%−15.8%, volume: 24.9%−34.2%). The environmental variation coefficients for volume in the three sites were greater than those for height and DBH (Fig. 4a). To identify the main environmental factors affecting growth, we collected meteorological data from the three sites from 2009 to 2014 and explored the correlation between clonal growth traits and environmental factors through Spearman's correlation and regression analysis (Fig. 4b; Supplementary Figs S1−S3). The results indicated that height, DBH, and volume responded to different environmental factors. The height was moderately positively correlated with temperature difference (TD) (0.36) and hargreaves climatic moisture deficit (CMD) (0.35), exhibiting a certain linear regression pattern with R^2^ (0.077) being the highest among all environmental parameters; DBH was moderately and significantly positively correlated with mean coldest month temperature (MCMT) (0.41) and moderately and significantly negatively correlated with degree-days below 18°C (DD < 18) (−0.39). These two parameters had the highest and fifth highest R² values, respectively, in regression analysis with DBH, at 0.116 and 0.066; Volume was moderately and positively correlated with TD (0.39) and CMD (0.31), with CMD showing the highest linear regression R² value of 0.135 (Supplementary Fig. S3). This suggests that TD, CMD, DD < 18, and MCMT are the main environmental factors affecting the growth of *C. bungei* (Fig. 4b, c). Additionally, we used the MRT to assess the combined effects of multiple environmental factors and found that TD is the primary environmental indicator for distinguishing the growth status of clonal trees. When TD is greater than 26.65 °C, and MCMT is greater than 0.1 °C, and CMD is greater than 520.5, the clones grow well; at the same time (Fig. 4d, Supplementary Fig. S4).

### Environmental drivers of G × E effects on the growth traits of clones

**Figure 5:**
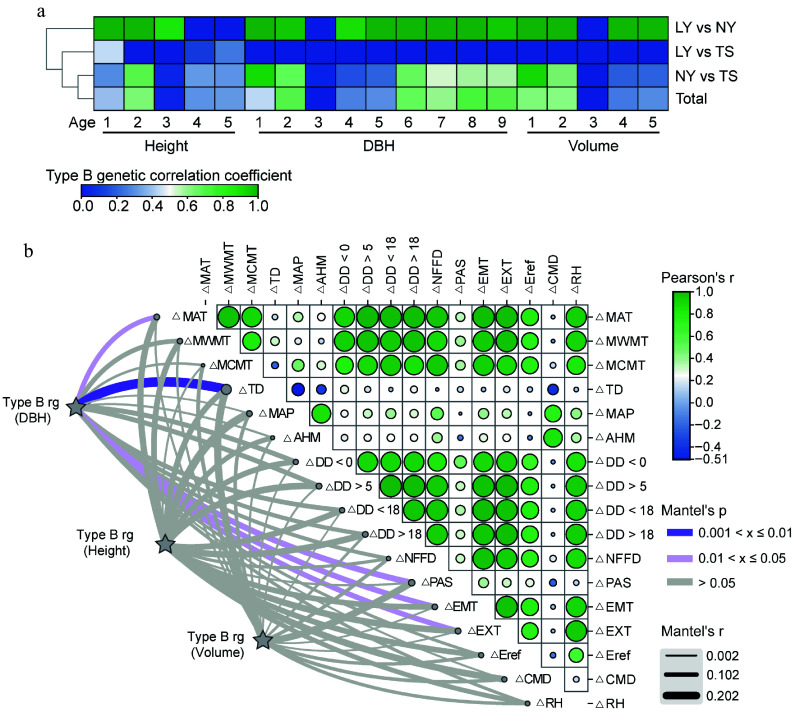
Analysis of the driving effect of environmental factors on G × E of growth traits. (a) B-type genetic correlations of multi-year growth traits of clones between different sites. (b) Mantel correlation analysis between B-type genetic correlations of growth traits and changes in environmental factors, with stronger correlations indicating a stronger driving effect of environmental factors on G × E effects of growth traits.

Environmental changes not only directly affect phenotypes but also drive G × E effects. The B-type genetic correlation results indicated that for clones in the LY and NY, except for the height at 4−5 years, DBH at 3 years, and volume at 3 years with genetic correlations close to 0, the rest had correlations around 1, suggesting that the G × E effects in these two sites are weak. The genetic correlations for most growth traits between LY and TS tended to be 0. The B-type genetic correlations for clone traits in NY and TS were similar to the combined estimates from the three sites, with the genetic correlation for height at 4−5 years around 0.3, for DBH at 6−9 years around 0.66−0.72, and volume at 4−5 years relatively low at 0.19−0.29. It is speculated that the larger geographical and environmental differences between the TS and the LY, NY are the reasons for the G × E effects (Fig. 5a). To further clarify the specific environmental drivers of G × E, the correlation strength between the changes in environmental factors and the B-type genetic correlations of traits was used as a measure, with stronger correlations indicating a stronger driving force. The results showed that changes in TD and precipitation as snow (PAS) had a significant impact on the genetic correlations for DBH and volume, with the impact on DBH reaching a significant level, while mean annual precipitation (MAP) and CMD had a greater impact on the genetic correlation for height. This indicates that TD, PAS, MAP, and CMD are the main environmental factors driving the growth of clones, with TD being the most critical factor (Fig. 5b).

### Selection of excellent clones under the strategy of site-appropriate tree species

**Figure 6:**
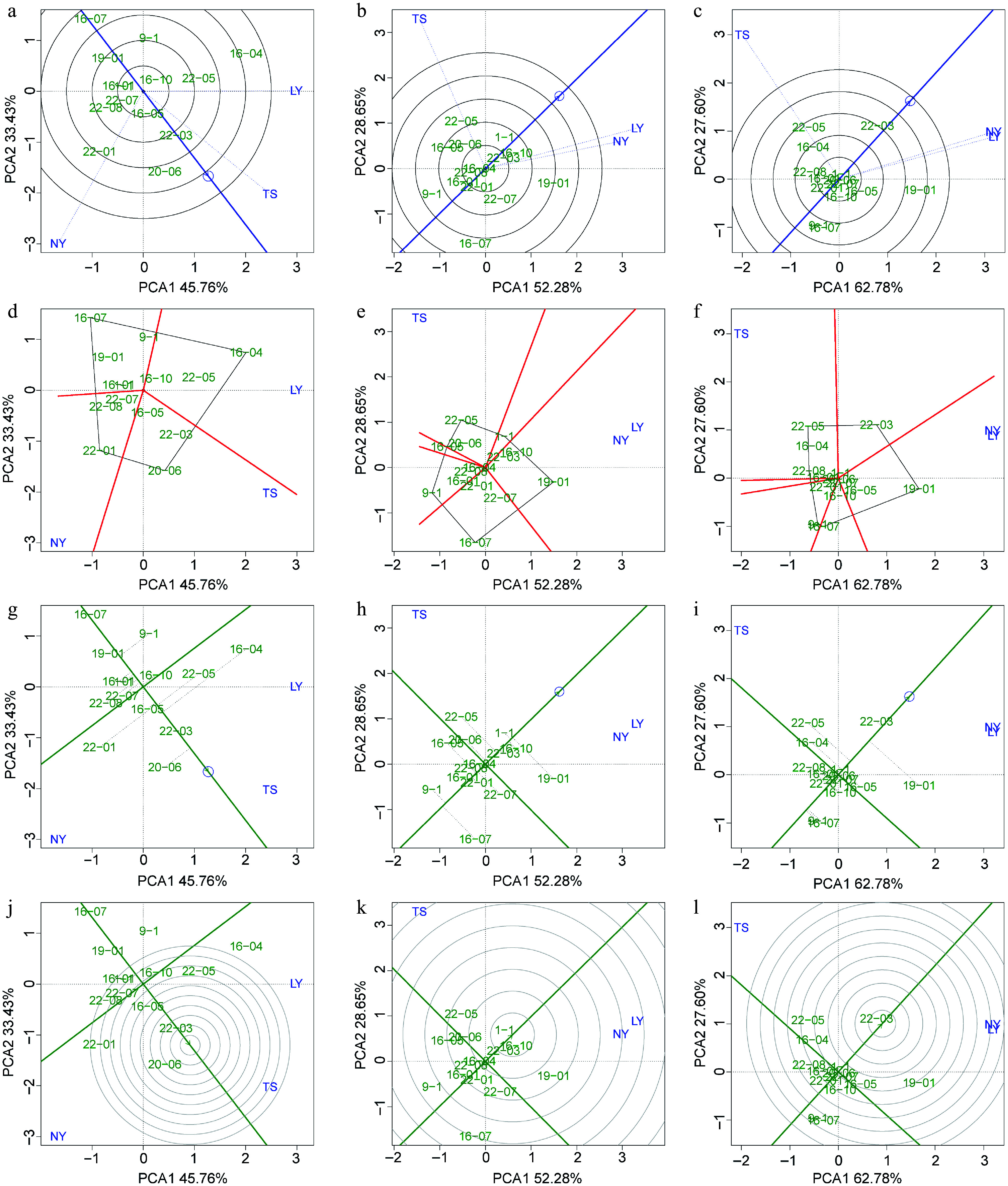
BLUP-GGE analysis for the tree height, DBH, and volume of clones. Green labels indicating clones and blue labels indicating sites. (a)−(c) 'Discrimination vs representativeness' analysis is shown the blue arrow line segment representing the average environmental axis, the length of the test point line segment indicates the discriminating power of the environment on the variety, and the angle between the line segment and the average environmental axis represents the degree of representativeness of the target environment; the smaller the angle, the stronger the representativeness. (d)−(f) The 'which and won where/what' module analysis is shown, where clones at the four vertices of the polygon represent the best clones within specific regions. (g)−(i) The 'mean vs stability' analysis is shown, with the arrowed line representing the environmental mean axis, the position of the arrow indicating the mean environmental value, and the perpendicular line from the clone to the mean environmental axis (gray dashed line) representing the stability of the clone's traits. The longer the perpendicular line, the worse the trait stability, and the closer the clone is to the arrow, the better the trait performance. (j)−(l) The 'rank genotype' analysis is presented, with concentric circles made relative to the mean environmental axis, with clones closer to the center indicating better mean values and stability for their traits.

To select excellent clones that are suitable for specific regions and have stable adaptability in multiple environments, we used BLUP-GGE to evaluate the environmental adaptability of clones with 5-year height, 9-year DBH, and 5-year volume as the target traits. The results of environmental discrimination and representativeness showed that for height as the target trait, the NY had the strongest discrimination power for varieties (the longest blue dashed line), and the TS was the most representative of the environment for *C. bungei* height growth (the smallest angle between the blue dashed line and the environmental average effect axis) (Fig. 6a). When DBH and volume were the targets, the TS had a stronger discrimination power for varieties, while the representativeness and discrimination power for variety growth in NY and LY were similar (Fig. 6b, c). The analysis of the best varieties in each site showed that clones 16-04, 22-01, and 20-06 were located at the vertices of the polygons in the LY, NY, and TS, respectively, indicating that they are the best clones for height growth in their respective region (Fig. 6d). Similarly, clone 19-01 was the largest DBH and volume clone in the LY and NY, and clone 22-05 was the largest DBH and volume clone in the TS (Fig. 6e, f). The analysis of the average and stability of variety traits indicated that, for height as the target, clone 20-06 was closest to the average effect center, indicating that its average height across multiple sites was the largest, and clone 22-03 was on the average effect axis (the shortest dashed line), indicating that it was the most stable in tree height across multiple environments (Fig. 6g). Similarly, clone 1-1 had the largest average DBH, and clone 16-04 had the best DBH stability (Fig. 6h). Clone 22-03 had the best average effect for volume and also had relatively strong stability (Fig. 6i). The results of the optimal clone screening showed that clone 22-03 was the excellent clone with both stability and high yield in tree height and volume (closest to the center of the concentric circles), and clone 1-1 was the excellent clone with both stability and high yield in DBH (Fig. 6j−l).

Finally, we assessed the genetic gain of traits for the excellent clones targeted at specific regions and integrated across multiple environments. Due to the small variation and repeatability of height in the LY, the height genetic gain of the excellent clone 16-04 was only 1.36%. In the TS, the genetic gain of the excellent clone 20-06 for height could reach 10.43%. The improvement potential for DBH was better than for height, with an estimated genetic gain of 13.94% for the excellent clone 19-01 in the NY. Volume had the highest genetic gain, and the multi-regional selection of clone 22-03 could achieve a relatively stable improvement effect, with an estimated genetic gain of 12.11%. The genetic gain for volume was higher when selecting the optimal clones in the LY, NY, and TS, with 18.13%, 21.46%, and 29.84% respectively (Table 1).

**Table 1:** The genetic gain of growth traits for excellent clones estimated from single-site and multi-site analyses.

Trait	5a height (m)					9a DBH (cm)					5a volume (cm^3^)			
Site	LY	NY	TS	Multiple sites		LY	NY	TS	Multiple sites		LY	NY	TS	Multiple sites
Excellent clone	16-04	22-01	20-06	22-03		19-01	19-01	22-05	1-1		19-01	19-01	22-05	22-03
Mean of excellent clone	9.01	7.96	9.34	8.51		15.03	14.55	12.64	13.09		0.0460	0.0366	0.0374	0.0362
Mean of population	8.63	7.40	8.18	8.07		13.35	12.37	10.44	12.06		0.0350	0.0260	0.0233	0.0271
Repeatability	0.31	0.67	0.73	0.49		0.49	0.79	0.41	0.72		0.58	0.52	0.49	0.36
Genetic gain (%)	1.36	5.11	10.43	2.70		6.12	13.94	8.56	6.22		18.13	21.46	29.84	12.11

## Discussion

### Genetic and environmental effects influencing the growth of *C. bungei* clones under different environments

Genetic variation forms the basis of genotype selection^[18]^. In this study, significant differences in growth were observed among different genotypes, and the genetic variation coefficient for volume was greater than that for height and DBH. It means that the evaluation and selection of excellent clones for volume are more favorable^[29]^. This finding is consistent with the results of studies on *Pinus masson*iana^[6]^, and *Liriodendron tulipifera*^[5]^. Another factor in the success of genetic improvement was whether the target traits had high heritability. The present study also found that after the growth of clones was stabilized at three sites, the repeatability of the DBH was stable at around 0.7, indicating that genetic improvement of DBH through the selection of superior genotypes has long-term reliability. However, this result still requires further verification, as the use of differentiated experimental designs across multiple sites may lead to model complexity and convergence issues, as well as biased parameter estimates for repeatability and genetic variances. Despite using plot means for modeling to simplify the linear model and standardize the estimation of genetic parameters.

Excluding genetic factors, temperature was the predominant factor influencing the growth of clones. MCMT has a weak negative effect on tree height but a significantly moderate positive effect on DBH. This difference is related to the higher phenological stability of tree height^[30]^.

Temperature can significantly alter the timing of cambial reactivation in the early growing season, affecting the radial differentiation of xylem cells^[31]^. Temperatures that are too low can even inhibit cambial activity^[32]^. Therefore, a higher MCMT may activate cambial differentiation in *C. bungei* trees earlier, promoting radial growth. We also found that DD < 18 exhibited a strong negative correlation with DBH, but Guo et al.^[33]^ showed that the radial growth of fir is significantly and positively correlated with accumulated temperatures between 7−9.5 °C. It is speculated that this correlation is related to the significant genetic background differences between broadleaf and coniferous trees. Thus, understanding the relationship between tree species growth and the environment is necessary before establishing cultivation measures, such as determining whether to provide adequate temperatures early in the growing season.

### The strength of G × E of *C. bungei* clones and its environmental drivers

In breeding, substantial G × E impacts directly influence the ranking of varieties, a phenomenon confirmed in many significant economic tree species, such as *Populus euramericana*^[34]^, and *P. massoniana*^[6]^. Therefore, the assessment of G × E is necessary. In addition to intensity, there may also be notable differences in G × E effects among traits under the same environmental conditions, which are related to the age effects and environmental response patterns of the traits^[5,23,35]^. This study also discovered that the G × E effects on tree height, DBH, and volume in *C. bungei* clones vary in response to environmental changes, underscoring the importance of tailored breeding strategies for different traits.

The G × E effects stem from significant environmental variations across geographical regions and years. Early research indicates that for a specific set of genotypes, the more diverse the environment, the greater the potential strength of G × E^[9]^. The elevation is considered a major driver of G × E because it is associated with a range of important bioclimatic factors, including rainfall, seasonality, and temperature changes^[10]^. For instance, the dry environment and short growing season caused by high elevation are the most important factors leading to G × E in the tree height and DBH of poplars^[35]^; the G × E in *P. radiata* is attributed to the heavy snow load resulting from high elevation^[11]^. This study also found, through B-type genetic correlation analysis, that there is a weaker genetic correlation and stronger G × E effect for growth traits of *C. bungei* between TS and LY or TS and NY, which has a significant elevation difference^[36]^. And we found that the TD caused by geographical environmental differences may be the main driver of the DBH G × E of *C. bungei* clones, which is similar to the finding that extreme maximum and minimum temperatures may drive the DBH G × E in *P. radiata*^[37]^.

### Combined selection of superior genotypes

There are many methods to assess G × E, and GGE biplot mapping has been widely applied in numerous forest tree G × E studies^[20,38]^ because it can simultaneously consider the stability and productivity of varieties and has strong visualization capabilities^[18,38]^. However, G × E is a complex effect, and in practical situations, linear models face issues with unequal error variances, which greatly reduces the reliability of the results. GGE analysis relies on trait means making it difficult for the method to effectively exclude the significant impact of environmental differences on the results.

To circumvent this factor, Cheng et al.^[20]^ proposed a method that combines trait BLUP values with the GGE model, called BLUP-GGE. This approach directly uses the genetic effect values of traits for analysis, making it more reliable. Therefore, this study used BLUP-GGE to assess the stability and yield of *C. bungei* clones. The results indicated that, with 5-year tree height as the improvement target, clones 16-04, 22-01, and 20-06 were the optimal clones for the LY, NY, and TS, respectively. With 9-year DBH and 5-year volume as targets, clone 19-01 was the optimal clone for the LY and NY, while clone 22-05 performed best in the TS. Considering both stability and productivity, clone 22-03 was identified as a clone with both high yield and stability in tree height and volumes, and clone 1-1 as a clone with both high yield and stability in DBH. This differs from the optimal clones at individual sites, highlighting the importance of implementing G × E effect-based breeding policies and selecting trees suitable for specific locations. Considering the limitations of linear models, in the future, estimating the effect values of SNPs through genomic sequencing to predict breeding values will help further reduce the impact of environmental errors and improve the accuracy of genetic evaluation.

## Conclusions

The phenotype of trees is determined by both genetics and environment. This study assessed the genetic, environmental, and G × E effect variations of *C. bungei* clone growth traits over multiple years in three different geographical environments using mixed linear models. It was found that there was significant genetic variation and moderate repeatability in the growth traits of clones, indicating a good foundation for genetic improvement through genotypic selection. The environmental effect was the most significant factor affecting clonal growth, with MCMT showing a strong positive correlation with DBH, and DD < 18 showing a negative correlation with DBH; TD and CMD were positively correlated with height and volume. Clones exhibited better growth characteristics when TD > 26.65 °C, MCMT > 0.1 °C, and CMD > 520.5. Additionally, the growth of clones showed a highly significant G × E interaction, with TD being the main environmental factor driving the G × E of clone DBH. Based on the BLUP values of clonal growth traits and using the BLUP-GGE method, clone 1-1 was selected as a high-yielding and stable clone for DBH, with a multi-site combined genetic gain of 6.22%, and clone 22-03 was selected as a high-yielding and stable clone for volume, with a multi-site combined genetic gain of 12.11%. The selected superior clones are expected to increase the productivity of *C. bungei* artificial forests and may be cultivated and promoted in multiple regions in the future.

## SUPPLEMENTARY DATA

Supplementary data to this article can be found online.

## Data Availability

All data generated or analyzed during this study are included in this published article and its supplementary information files.

## References

[1] (2015). Evaluating testing strategies for plant breeding field trials: redesigning a CIMMYT international wheat nursery. Crop Science.

[2] (2016). Analysis of the genetic variation in growth, ecophysiology, and chemical and metabolomic composition of wood of *Populus trichocarpa* provenances. Tree Genetics & Genomes.

[3] (2011). Leaf area index development in temperate oak and beech forests is driven by stand characteristics and weather conditions. Trees.

[4] (2016). Adaptability, stability, productivity and genetic parameters in slash pine second-generation families in early age. Silvae Genetica.

[5] (2022). Growth performance and G × E interactions of *Liriodendron tulipifera* half-sib families across ages in eastern China. European Journal of Forest Research.

[6] (2021). Genetic parameters and genotype by environment interactions influencing growth and productivity in Masson pine in east and central China. Forest Ecology and Management.

[7] (2021). Genotype by environment interaction analysis of growth of *Picea koraiensi*s families at different sites using BLUP-GGE. New Forests.

[8] (2021). Genotype-environment interaction and stability of fiber properties and growth traits in triploid hybrid clones of *Populus tomentosa*. BMC Plant Biology.

[9] (2015). Genetic parameters and clone by environment interactions for growth and foliar nutrient concentrations in radiata pine on 14 widely diverse New Zealand sites. Tree Genetics & Genomes.

[10] (2011). Genotype by environment interactions for *Pinus radiata* in New South Wales, Australia. Tree Genetics & Genomes.

[11] (2005). Genotype by environment interactions in an Australia-wide radiata pine diallel mating experiment: implications for regionalized breeding. Forest Science.

[12] (2000). Study on selection of *Aluns cremastogyne* provenance/family-analysis of growth adaptation and genetic stability. Scientia Silvae Sinicae.

[13] (2022). A critical review on the principles and procedures for cultivar development and evaluation. Acta Agronomica Sinica.

[14] (2017). Genotype by environment interactions in forest tree breeding: review of methodology and perspectives on research and application. Tree Genetics & Genomes.

[15] (1963). The analysis of adaptation in a pant breeding programme. Australian Journal of Agricultural Research.

[16] (1977). Genetic correlation as a concept for studying genotypeenvironment interaction in forest tree breeding. Silvae Genetica.

[17] (2001). GGEbiplot—a windows application for graphical analysis of multienvironment trial data and other types of two-way data. Agronomy Journal.

[18] (2007). Genotype×environment interactions in selected loblolly and slash pine plantations in the Southeastern United States. Forest Ecology and Management.

[19] (2016). AMMI model for yield estimation in multi-environment trials: a comparison to BLUP. Agriculture and Agricultural Science Procedia.

[20] (2018). Forestry multi-environment trial analysis based on BLUP and GGE biplot. Journal of Northwest A&F University (Natural Science Edition).

[21] (2010). Optimal use of biplots in analysis of multi-location variety test data. Acta Agronomica Sinica.

[22] (2022). G×E analysis of early growth traits of *Populus deltoides* in east China by using BLUP-GGE. Forests.

[23] (2019). Genetic variation of growth traits and genotype-by-environment interactions in clones of *Catalpa bungei* and *Catalpa fargesii* f. *duclouxii*. Forests.

[24] (2017). ClimateAP: an application for dynamic local downscaling of historical and future climate data in Asia Pacific. Frontiers of Agricultural Science and Engineering.

[b25] 25Borcard D, Gillet F, Legendre P. 2018. Numerical ecology with R. New York: Springer. xv, 435 pp.

[26] (2002). Multivariate regression trees: a new technique for modeling species-environment relationships. Ecology.

[b27] 27Therneau TM, Atkinson B, Ripley B, Oksanen J. 2014. mvpart: multivariate partitioning. R package version 1.6-2. Retrieved from https://CRAN.R-project.org/package=mvpart

[28] (2021). Using a multivariate regression tree to analyze trade-offs between ecosystem services: application to the main cropping area in France. Science of The Total Environment.

[29] (2004). Genetic variation in growth traits in a *Quercus robur* L. open pollinated progeny test of the slavonian provenance. Silvae Genetica.

[30] (2024). Above- and belowground phenology responses of subtropical Chinese fir (*Cunninghamia lanceolata*) to soil warming, precipitation exclusion and their interaction. Science of The Total Environment.

[31] (2007). Regular cambial activity and xylem and phloem formation in locally heated and cooled stem portions of Norway spruce. Wood Science and Technology.

[32] (2003). The importance of early summer temperature and date of snow melt for tree growth in the Siberian Subarctic. Trees.

[33] (2019). Effects of accumulated and threshold temperatures on the radial growth of *Abies faxonian*a in the alpine timberline, Western Sichuan Plateau. Acta Ecologica Sinica.

[34] (2021). Analysis of the genotype interaction of four-year-old *Populus euramericana* using the BLUP-GGE technique. Forests.

[35] (2011). Pattern of genotype–environment interaction in *Picea glauca* (Moench) Voss in Alberta, Canada. Annals of Forest Science.

[36] (2017). Patterns of additive genotype-by-environment interaction in tree height of Norway spruce in southern and central Sweden. Tree Genetics & Genomes.

[37] (2015). Drivers of genotype by environment interaction in radiata pine as indicated by multivariate regression trees. Forest Ecology and Management.

[38] (2007). GGE Biplot vs. AMMI analysis of genotype-by-environment data. Crop Science.

