# Supplementary material for: Growth adaptability and stability in Catalpa bungei clones: the role of genetics and environment
Source: For Res (Fayettev). 2025 Jan 22;5:e002. doi: 10.48130/forres-0025-0003 (PMC11870305; doi:10.48130/forres-0025-0003)
Supplement: Supplementary file 1 — Supplementary data to this article can be found online. [file forres-0025-0003-S1.zip › 10.48130_forres-0025-0003-Suppl-FigureS2.pdf]

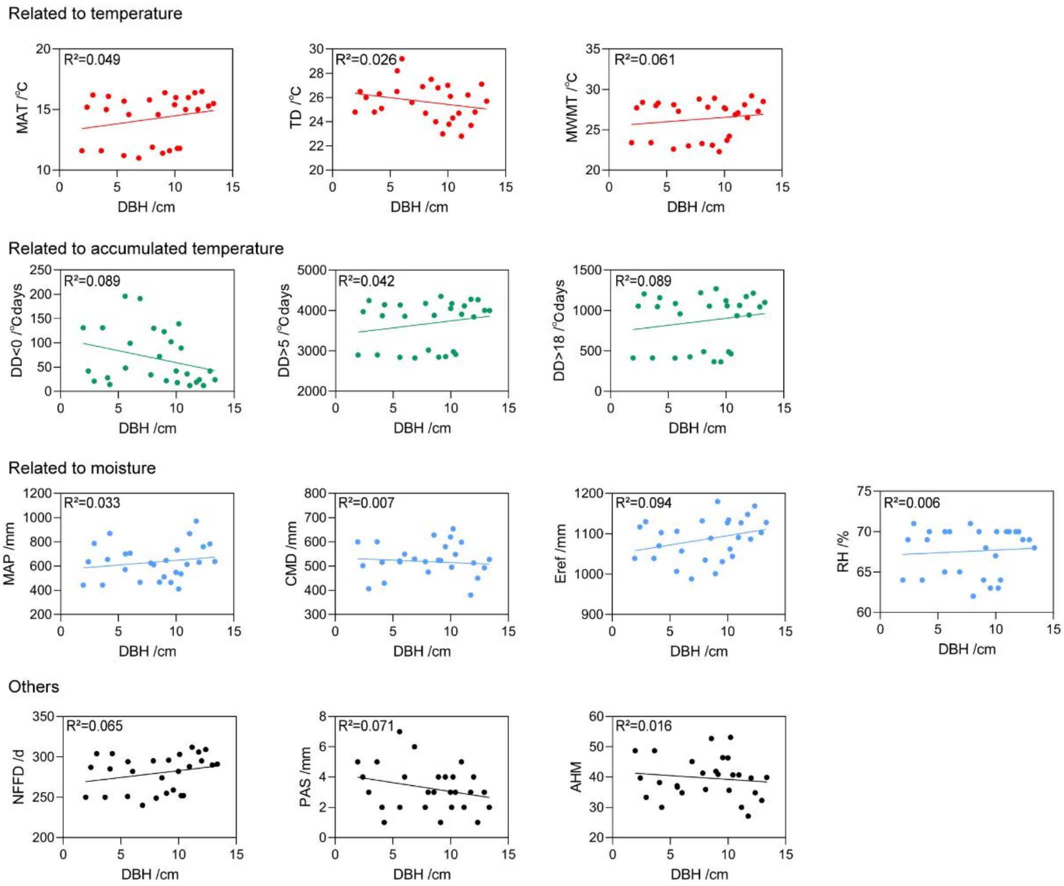

Figure S2 Linear regression analysis of DBH with environmental factors
