# Supplementary material for: Growth adaptability and stability in Catalpa bungei clones: the role of genetics and environment
Source: For Res (Fayettev). 2025 Jan 22;5:e002. doi: 10.48130/forres-0025-0003 (PMC11870305; doi:10.48130/forres-0025-0003)
Supplement: Supplementary file 1 — Supplementary data to this article can be found online. [file forres-0025-0003-S1.zip › 10.48130_forres-0025-0003-Suppl-TableS1.pdf]

Table S1 Genetic background of *Catalpa bungei* clones

Clones	Hybrid type	Parental origin
6--05	*catalpa bungei* hybridization	10-2×2-1
9--05	*catalpa bungei* hybridization	2-2×6-8
19--27	*catalpa bungei* hybridization	5-8×6-8
19--12	*catalpa bungei* hybridization	5-8×6-8
19--01	*catalpa bungei* hybridization	5-8×6-8
12--13	*catalpa bungei* hybridization	6-1×5-8
12--09	*catalpa bungei* hybridization	6-1×5-8
20--06	*catalpa bungei* hybridization	6-1×6-8
20--02	*catalpa bungei* hybridization	6-1×6-8
20--01	*catalpa bungei* hybridization	6-1×6-8
13--06	*catalpa bungei* hybridization	6-2×6-8
13--05	*catalpa bungei* hybridization	6-2×6-8
16--10	*catalpa bungei* hybridization	6-2×8-1
16--07	*catalpa bungei* hybridization	6-2×8-1
16--05	*catalpa bungei* hybridization	6-2×8-1
16--04	*catalpa bungei* hybridization	6-2×8-1
16--01	*catalpa bungei* hybridization	6-2×8-1
18--09	*catalpa bungei* hybridization	6-8×5-8
17--06	*catalpa bungei* hybridization	6-8×6-7
17--05	*catalpa bungei* hybridization	6-8×6-7
1--1	*catalpa bungei* hybridization	6-8×*Catalpa longicarpa*
9--1	*catalpa bungei* hybridization	8-5×6-7
7--01	*catalpa bungei* hybridization	9-3×2-1
23--05	*catalpa bungei*	2-3 Free pollination
22--10	*catalpa bungei*	4038 Free pollination
22--08	*catalpa bungei*	4038 Free pollination
22--07	*catalpa bungei*	4038 Free pollination
22--05	*catalpa bungei*	4038 Free pollination
22--03	*catalpa bungei*	4038 Free pollination
22--01	*catalpa bungei*	4038 Free pollination
21--03	*catalpa bungei*	6-7 Free pollination
21--02	*catalpa bungei*	6-7 Free pollination
