# Supplementary material for: Growth adaptability and stability in Catalpa bungei clones: the role of genetics and environment
Source: For Res (Fayettev). 2025 Jan 22;5:e002. doi: 10.48130/forres-0025-0003 (PMC11870305; doi:10.48130/forres-0025-0003)
Supplement: Supplementary file 1 — Supplementary data to this article can be found online. [file forres-0025-0003-S1.zip › 10.48130_forres-0025-0003-Suppl-TableS2.pdf]

Table S2 Multi-location variance analysis of clone growth

Traits	Age	Site				Clone			
		Mean Square	F value	P value	df	Mean Square	F value	P value	df
Height	1	26.053	348.035	< 2e-16	2	0.229	3.053	5.19E-07	31
	2	9.396	43.219	< 2e-16	2	0.834	3.836	6.91E-10	31
	3	77.400	247.384	< 2e-16	2	1.120	3.591	5.41E-09	31
	4	94.850	256.037	< 2e-16	2	1.450	3.924	3.32E-10	31
	5	50.810	114.796	< 2e-16	2	2.060	4.645	8.32E-13	31
DBH	1	32.090	281.186	< 2e-16	2	0.210	1.828	0.005990	31
	2	14.025	81.975	< 2e-16	2	0.729	4.259	1.99E-11	31
	3	5.227	7.885	0.000465	2	3.332	5.027	4.02E-14	31
	4	75.310	60.229	< 2e-16	2	4.170	3.334	4.92E-08	31
	5	98.400	52.665	< 2e-16	2	5.070	2.713	0.00000761	31
	6	104.300	53.143	< 2e-16	2	7.780	3.967	2.43E-10	31
	7	170.250	82.641	< 2e-16	2	9.760	4.737	4.23E-13	31
	8	189.280	83.613	< 2e-16	2	9.890	4.371	8.80E-12	31
	9	239.020	104.269	< 2e-16	2	9.740	4.249	2.42E-11	31
Volume	1	2.26E-05	312.182	< 2e-16	2	1.33E-07	1.831	0.00597	31
	2	5.27E-05	73.094	< 2e-16	2	3.02E-06	4.184	3.73E-11	31
	3	2.42E-04	35.453	1.82E-14	2	2.69E-05	3.938	3.29E-10	31
	4	1.89E-03	67.271	< 2e-16	2	8.29E-05	2.953	0.00000114	31
	5	3.66E-03	49.81	< 2e-16	2	1.97E-04	2.684	0.00000967	31

Continue to Table S2

Traits	Age	Site × Clone				Error	
		Mean Square	F value	P value	df	Mean Square	df
Height	1	0.141	1.880	0.0013	44	0.075	280
	2	0.336	1.545	0.0199	44	0.217	290
	3	1.050	3.371	3.86E-10	44	0.310	290
	4	0.910	2.456	5.09E-06	44	0.370	290
	5	1.300	2.945	3.45E-08	44	0.440	288
DBH	1	0.150	1.282	0.1206	44	0.110	290
	2	0.255	1.489	0.0303	44	0.171	290
	3	3.073	4.636	1.43E-15	43	0.663	283
	4	2.470	1.974	0.000605	43	1.290	284
	5	2.990	1.602	0.0128	44	1.870	290
	6	2.780	1.417	0.0504	44	1.960	287
	7	3.690	1.793	0.0027	44	2.060	284
	8	3.270	1.444	0.0421	44	2.260	284
	9	3.320	1.448	0.0409	44	2.290	284
Volume	1	8.50E-08	1.17	0.22625	44	7.20E-08	280
	2	1.13E-06	1.57	0.0164	44	7.20E-07	290
	3	2.87E-05	4.2	1.19E-13	43	6.83E-06	283
	4	5.22E-05	1.861	0.00164	43	2.81E-05	283
	5	1.26E-04	1.722	0.00485	44	7.30E-05	288
